# Conditional Inactivation of TNFα-Converting Enzyme in Chondrocytes Results in an Elongated Growth Plate and Shorter Long Bones

**DOI:** 10.1371/journal.pone.0054853

**Published:** 2013-01-18

**Authors:** Kenta Saito, Keisuke Horiuchi, Tokuhiro Kimura, Sakiko Mizuno, Masaki Yoda, Hideo Morioka, Haruhiko Akiyama, David Threadgill, Yasunori Okada, Yoshiaki Toyama, Kazuki Sato

**Affiliations:** 1 Department of Orthopedic Surgery, School of Medicine, Keio University, Tokyo, Japan; 2 Department of Anti-Aging Orthopedic Research, School of Medicine, Keio University, Tokyo, Japan; 3 Department of Pathology, School of Medicine, Keio University, Tokyo, Japan; 4 Department of Orthopedics, Kyoto University, Kyoto, Japan; 5 Department of Genetics, North Carolina State University, Raleigh, North Carolina, United States of America; INSERM U1059/LBTO, Université Jean Monnet, France

## Abstract

TNFα-converting enzyme (TACE) is a membrane-bound proteolytic enzyme with essential roles in the functional regulation of TNFα and epidermal growth factor receptor (EGFR) ligands. Previous studies have demonstrated critical roles for TACE *in vivo*, including epidermal development, immune response, and pathological neoangiogenesis, among others. However, the potential contribution of TACE to skeletal development is still unclear. In the present study, we generated a *Tace* mutant mouse in which *Tace* is conditionally disrupted in chondrocytes under the control of the *Col2a1* promoter. These mutant mice were fertile and viable but all exhibited long bones that were approximately 10% shorter compared to those of wild-type animals. Histological analyses revealed that *Tace* mutant mice exhibited a longer hypertrophic zone in the growth plate, and there were fewer osteoclasts at the chondro-osseous junction in the *Tace* mutant mice than in their wild-type littermates. Of note, we found an increase in osteoprotegerin transcripts and a reduction in *Rankl* and *Mmp-13* transcripts in the TACE-deficient cartilage, indicating that dysregulation of these genes is causally related to the skeletal defects in the *Tace* mutant mice. Furthermore, we also found that phosphorylation of EGFR was significantly reduced in the cartilage tissue lacking TACE, and that suppression of EGFR signaling increases osteoprotegerin transcripts and reduces *Rankl* and *Mmp-13* transcripts in primary chondrocytes. In accordance, chondrocyte-specific abrogation of *Egfr in vivo* resulted in skeletal defects nearly identical to those observed in the *Tace* mutant mice. Taken together, these data suggest that TACE-EGFR signaling in chondrocytes is involved in the turnover of the growth plate during postnatal development via the transcriptional regulation of osteoprotegerin, *Rankl*, and *Mmp-13*.

## Introduction

Endochondral ossification is essential not only for the formation of cartilage models of future bones during embryogenesis but also for the longitudinal growth of the long bones during postnatal development. This process occurs in the growth plate (epiphyseal plate) where resting chondrocytes differentiate sequentially into proliferative, pre-hypertrophic and hypertrophic chondrocytes. Histologically, the growth plate can be divided into four distinct layers: the resting zone, proliferating zone, maturing zone, and hypertrophic zone. In the hypertrophic zone, differentiated chondrocytes undergo apoptosis, and the cartilage matrix becomes calcified. The calcified matrix is subsequently invaded by newly formed vessels and absorbed by osteoclasts. Not surprisingly, many growth factors and transcription factors are involved in these processes, and the expression patterns of these genes are spatially and temporally regulated to achieve normal skeletal growth [1]–[4].

TNFα-converting enzyme (TACE), also known as ADAM17 (a disintegrin and metalloprotease 17), is a membrane-bound proteolytic enzyme that is involved in the proteolytic release of the extracellular domain of the membrane-bound precursor of TNFα [5], [6]. The proteolytic release of the extracellular domains of membrane-bound proteins, which is also referred to as “ectodomain shedding”, has emerged as a crucial posttranslational regulator of the function and availability of membrane-bound proteins. Although TACE was originally identified as a pro-TNFα “sheddase”, and both TNFα- and TNF receptor-deficient mice are viable [7], [8], TACE deficiency in mice was unexpectedly found to be embryonic lethal with defects resembling those found in epidermal growth factor receptor (EGFR)-deficient mice [9]–[12]. Subsequent studies revealed that TACE has an unusually wide range of target molecules, including EGFR ligands (such as TGFα and heparin-binding EGF-like growth factor), CD44, Kit ligand, and L-selectin [9], [13]–[16], suggesting that TACE is a central component of the ectodomain shedding pathway *in vivo*. However, the early lethality of TACE-deficient mice has hampered the analysis of TACE functions in postnatal development and adult homeostasis [9], [17]. To circumvent this issue, we generated TACE conditional knockout mice and showed that the conditional inactivation of TACE resulted in a very complex phenotype in adult animals [17], [18]. Mutant mice in which *Tace* was abolished in various tissues under the control of the *Sox9* promoter exhibited defects in the hematopoietic system, skin, and fur, as well as severe growth retardation and low bone mass, indicating that TACE is involved in postnatal skeletal development [18]. However, due to the complexity of the defects observed in these mutant mice, the contribution of TACE activity to skeletal development could not be determined precisely.

In the present study, we aimed to clarify the roles of TACE in postnatal skeletal development. We generated a mutant line in which *Tace* was specifically disrupted in chondrocytes using *Col2a1* promoter-driven *cre*-transgenic mice [19]. Mice lacking TACE in chondrocytes did not show any overt defects, however, their long bones were approximately 10% shorter than those of wild-type animals. Histological analyses revealed a significantly elongated hypertrophic zone in the growth plate and a lower number of osteoclasts at the chondro-osseous junction, indicating that the elongation of the hypertrophic zone was most likely caused by a defective resorption of calcified matrix. In support of this notion, quantitative PCR analysis revealed an increase in the expression of osteoprotegerin (OPG) transcripts, and a decrease in the expression of receptor activator of nuclear factor kappa-B ligand (RANKL) and *Mmp-13* transcripts, indicating that aberrant expression of these genes is casually related to the skeletal defects in *Tace* mutant mice. Furthermore, we found that phosphorylation of EGFR was significantly reduced in the cartilage tissues lacking TACE, and that inhibition of EGFR signaling in primary chondrocytes induces *Opg* transcripts, whereas suppresses those of *Rankl* and *Mmp-13*. In accordance, mutant mice with conditional ablation of *Egfr* in chondrocytes exhibited skeletal defects similar to those observed in the *Tace* mutant mice. Collectively, these data suggest that the TACE-EGFR axis plays a critical role in the turnover of calcified cartilage matrix through the regulation of *Rankl*, *Opg*, and *Mmp-13* transcription, thus affecting osteoclast recruitment and matrix degradation.

## Results

### Chondrocyte-specific inactivation of TACE results in shorter long bones

**Figure 1:**
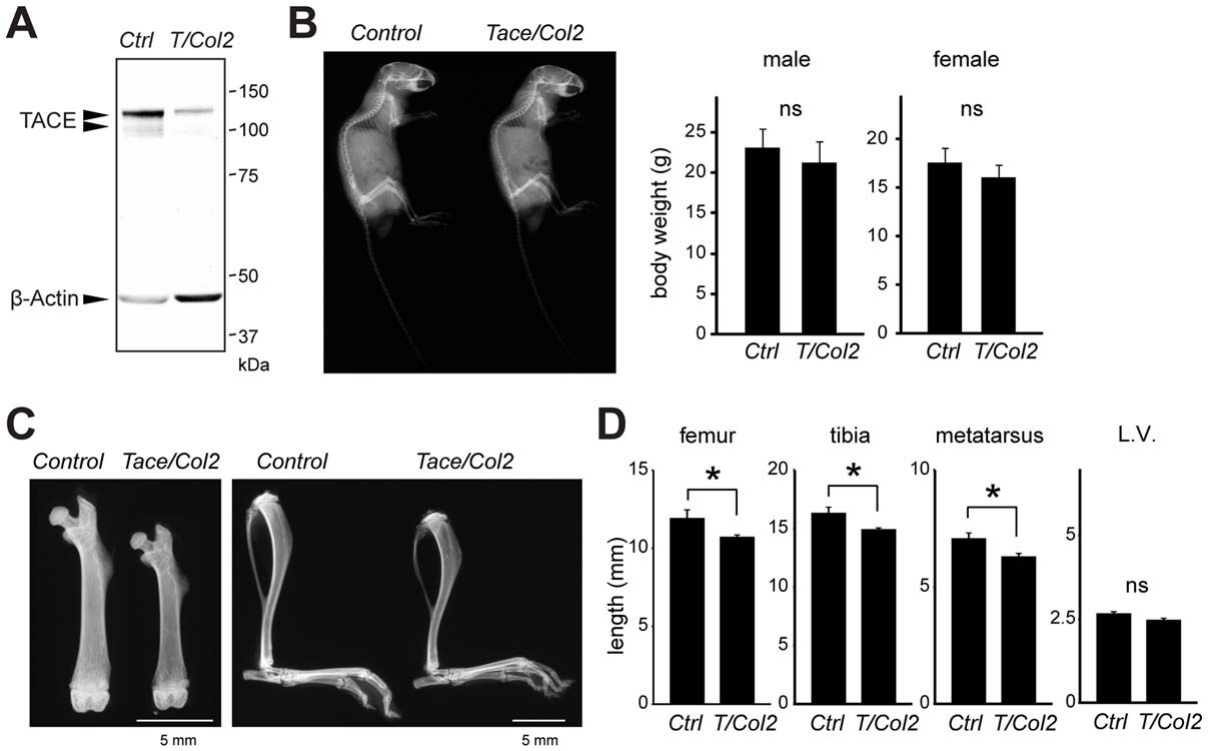
*Tace/Col2* mice exhibit shorter long bones. (A) Western blot analysis of TACE and β-Actin in primary chondrocytes harvested from *Control* (*Ctrl*) or *Tace/Col2* (*T/Col2*) mice. (B) Whole body X-rays reveal no overt abnormalities in the skeletal systems of 8-week-old *Tace/Col2* mice compared with the *Control* animals (left panel). There was no significant difference in the body weights of the 8-week-old *Control* and *Tace/Col2* mice (right panel). (C) X-rays of the lower limbs of 8-week-old *Tace/Col2* and *Control* mice. (D) Comparison of the length of the femur, tibia, third metatarsus, and the sixth lumbar vertebra (L.V.) between 8-week-old *Tace/Col2* mice and *Control* mice (right panel). *, *p*<0.05. ns, not significant. The results are expressed as the mean ± SD of at least three mice in each group.

To understand the potential contribution of TACE in the skeletal development, we generated a mutant line in which TACE was specifically inactivated in chondrocytes by crossing *Tace ^flox/flox^* mice with a transgenic line in which the transcription of *cre* was driven by a chondrocyte-specific *Col2a1* promoter [19] (*Tace ^flox/flox^/Col2a1-cre*, henceforth referred to as *Tace/Col2* mice). As shown in Fig. 1A, primary chondrocytes from *Tace/Col2* newborn mice expressed very low levels of TACE protein. *Tace/Col2* mice were fertile and viable. The offspring of *Tace/Col2* and *Tace ^flox/flox^* mice crosses were born in the expected Mendelian ratios (data not shown). *Tace/Col2* mice did not exhibit overt skeletal defects or growth retardation (Fig. 1B, and data not shown); however, we found that the length of the long bones was approximately 10% shorter in mutant mice compared to *Tace^flox/flox^* counterparts (henceforth referred to as *Control*) at 8 weeks of age (Fig. 1C and D). The height of the vertebrae was slightly lower in *Tace/Col2* mice compared to *Control* mice, but the difference was not statistically significant. These observations indicate that TACE expressed in chondrocytes is involved in the longitudinal growth of long bones, and show that lack of TACE activity in chondrocytes results in shorter long bones.

### *Tace/Col2* mice exhibit an elongated growth plate during development

**Figure 2:**
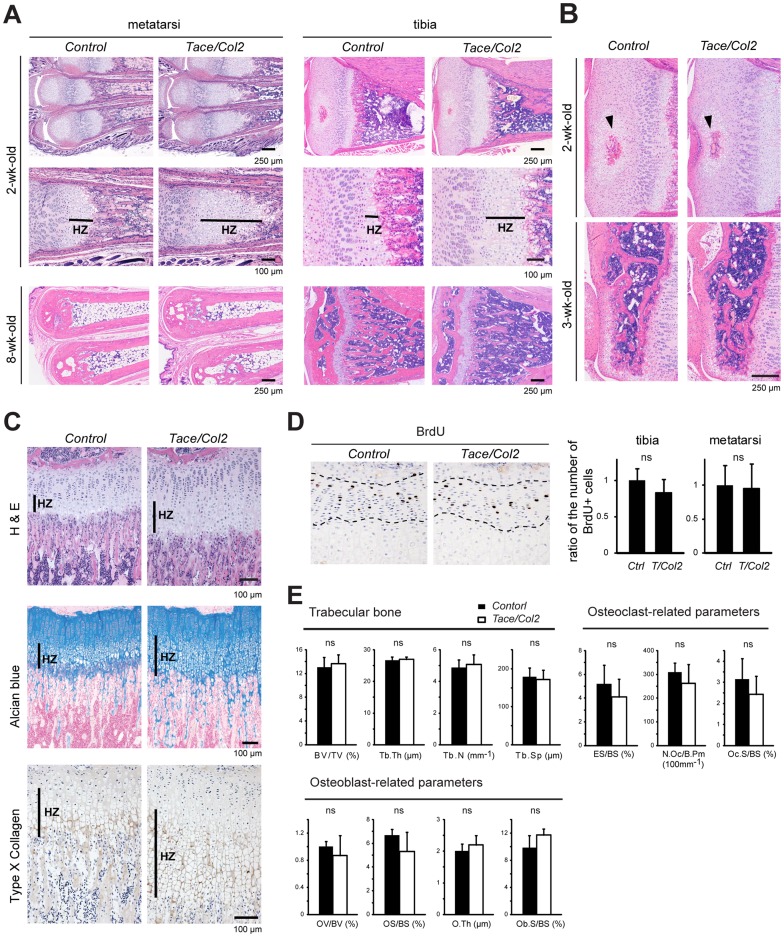
Histological and histomorphometric analysis of the long bones of *Control* and *Tace/Col2* mice. (A) Histological analysis of the metatarsi and tibia (2-week-old, upper panels, and 8-week-old, lower panels, respectively) from *Tace/Col2* and *Control* mice. HZ, hypertrophic zone. (B) Histological analysis of the secondary ossification center of the tibia (2-week-old, upper panels, and 3-week-old, lower panels, respectively) from *Tace/Col2* and *Control* mice. Arrowheads indicate the secondary ossification center. (C) Sections of tibiae from 3-week-old *Tace/Col2* and *Control* mice stained with alcian blue (upper panels) or immunostained with anti-type X collagen antibody. (D) Immunolocalization of BrdU-incorporated chondrocytes in the proliferating zone (outlined by broken lines) of the tibia harvested from *Control* and *Tace/Col2* mice (left panels). The ratio of the number of BrdU-positive chondrocytes in the proliferating zone of the tibia and metatarsus in *Control* and *Tace/Col2* mice (right panels). The average number of BrdU-positive cells from four *Control* sections is set as 1. (E) Histomorphometric analysis of the femurs of 8-week-old *Control* and *Tace/Col2* mice. BV/TV, bone volume/total volume; Tb. Th., trabecular thickness; Tb. N, trabecular number; Tb. Sp, trabecular separation; OV/BV, osteoid volume/bone volume; OS/BS, osteoid surface/bone surface; O. Th, osteoid thickness; Ob.S/BS, osteoblast surface/bone surface; ES/BS, eroded surface/bone surface; N.Oc/B.Pm, osteoclast numbers/osteoclast perimeter; Oc.S/BS, osteoclast surface/bone surface. ns, not significant. The results are expressed as the mean ± SD of at least 4 mice in each group.

Endochondral ossification in the growth plate is predominantly responsible for the longitudinal growth of long bones. We further examined the potential developmental defect in the growth plate of *Tace/Col2* mice. As shown in Fig. 2A, we found that the hypertrophic zone in the growth plate of *Tace/Col2* mice was significantly elongated compared to that in *Control* mice. The elongation of the growth plate is transient (most apparent at around 2 weeks of age) and becomes less obvious as the mice reach maturity. On the other hand, there was no significant difference in the timing of vascular invasion into the secondary ossification center between *Control* and *Tace-Col2* mice (Fig. 2B). Given these observations, we asked whether the phenotype in the growth plate was derived from defects in differentiation or a decrease in chondrocyte proliferation in the *Tace/Col2* mice. Because the growth plate in adult *Tace/Col2* mice appeared normal, we used younger mice for the analysis of the growth plate. As shown in Fig. 2C, we found no obvious difference in the staining pattern of alcian blue, indicating that there is no major difference in the localization of proteoglycans between *Control* and *Tace/Col2* mice. The expression pattern of type-X collagen appeared similar between *Control* and *Tace/Col2* mice, except that the type-X collagen-stained area was larger in the *Tace/Col2* mice, due to the elongation in the hypertrophic zone. These observations indicate that the development of chondrocytes is not, at least up to this particular stage, affected by the lack of TACE activity. Next, we examined the proliferation rate of the cells in the proliferating zone by 5-bromodeoxyuridine (BrdU) labeling, but there was no significant difference in the number of BrdU-positive cells between the *Control* and *Tace/Col2* mice (Fig. 2D). TUNEL assay of the cartilage sections also showed no obvious difference between *Control* and *Tace/Col2* mice (data not shown). In addition, an analysis of the femur revealed no difference in any of the histomorphometric parameters between the *Control* and *Tace/Col2* mice, suggesting that bone homeostasis is maintained in *Tace/Col2* adult animals (Fig. 2E). Taken together, these data show that a lack of TACE activity in chondrocytes does not severely affect the differentiation or proliferation of chondrocytes.

### *Tace/Col2* mice have fewer TRAP-positive multinucleated cells at the chondro-osseous junction during postnatal development

**Figure 3:**
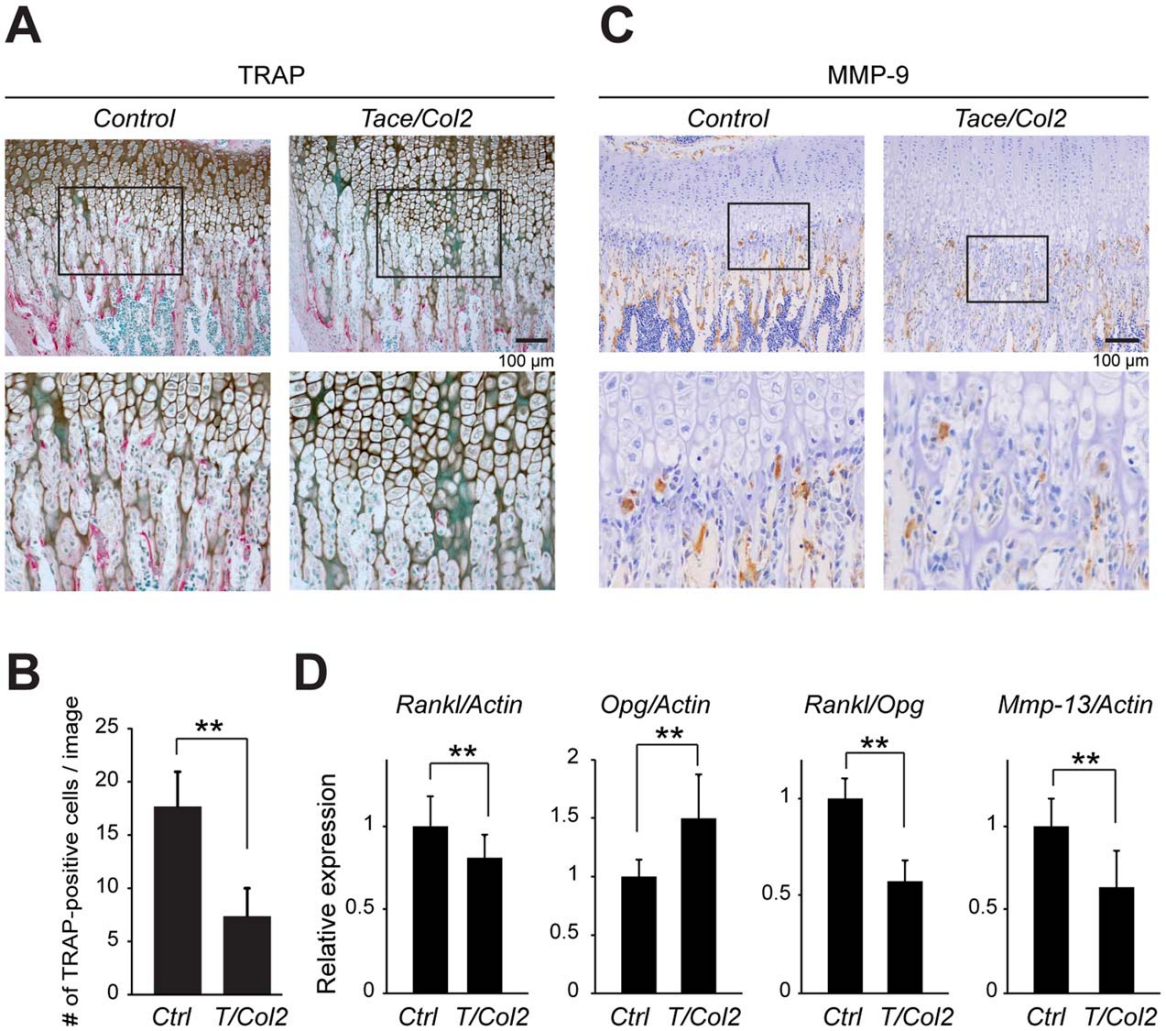
Fewer osteoclasts at the chondro-osseous junction of the growth plate in *Tace/Col2* mice. Tibia sections from 3-week-old *Control* and *Tace/Col2* mice stained for TRAP (A) or MMP-9 (C). The lower panels show high-magnification images of the boxed areas in the upper panels. (B) The number of TRAP-positive cells adjacent to the chondro-osseous junction per image in 3-week-old *Control* and *Tace/Col2* mice. The results are expressed as the mean ± SD of at least three mice (two sections from each mouse) in each group. (D) Quantitative analysis of the levels of *Rankl*, *Opg*, and *Mmp-13* transcripts in the cartilage tissues collected from *Control* and *Tace/Col2* mice. The results are expressed as the mean ± SD of at three independent experiments. **, *p*<0.005.

A close examination of the hematoxylin and eosin-stained sections of the metatarsi and tibiae revealed fewer multinucleated osteoclasts at the chondro-osseous junction in *Tace/Col2* mice than in *Control* animals (data not shown). In contrast, there was no apparent difference in the distribution of osteoclasts on the secondary spongiosa (data not shown), which is consistent with the results of the histomorphometric analysis (osteoclast-related parameters in Fig. 2E). To confirm this observation, we stained the sections for tartrate-resistant acid phosphatase (TRAP), an osteoclast marker, and evaluated the number and distribution of osteoclasts in the developing bone. As expected, there were fewer TRAP-positive multinucleated cells at the chondro-osseous junction in *Tace/Col2* mice than in *Control* mice (Fig. 3A and B) [20]. Immunostaining of MMP-9, a matrix metalloprotease that is highly expressed in osteoclasts, was consistent with the TRAP-staining (Fig. 3C). These histological analyses indicate that a lack of TACE activity in chondrocytes leads to a defect in the recruitment of osteoclasts to the calcified cartilage, resulting in a delay in the absorption of the calcified matrix in the hypertrophic zone. In accordance, we also observed more remnant cartilage tissue in the primary spongiosa in *Tace/Col2* mice than in *Control* animals (Fig. 2C, Alcian blue), suggesting that absorption of the cartilage matrix is not as efficient in the mutant mice as in *Control* animals. Based on these observations, we next asked whether the expression of genes involved in osteoclast activation and development, specifically *Rankl* and *Opg*, were deregulated in cartilage tissues lacking TACE. RANKL is an indispensable regulator of osteoclastogenesis and osteoclast activity, whereas OPG is a potent inhibitor of RANKL [21], [22]. We collected the cartilage tissues from 1-week-old *Tace/Col2* and *Control* mice, and analyzed the transcripts for these genes. As shown in Fig. 3D, we found a decrease in *Rankl* and an increase in *Opg* transcripts. As a result, the *Rankl*/*Opg* expression ratio was significantly reduced in the *Tace/Col2* cartilage compared with that in the wild-type control, indicating that the *Tace/Col2* cartilage is less potent in recruiting or stimulating osteoclast activity. In addition, we found that the transcripts for *Mmp-13*, a critical enzyme for cartilage matrix degradation during postnatal growth [23], [24], were also significantly reduced in the *Tace/Col2* cartilage. These observations suggest that the abrogation of TACE in cartilage tissues results in a decrease in osteoclast recruitment and MMP-13 expression, and consequently leads to a delay in the turnover of calcified cartilage matrix in the hypertrophic zone.

### Inhibition of EGFR signaling in chondrocytes decreases the ratio of *Rankl*/*Opg* and *Mmp-13* transcript expression

**Figure 4:**
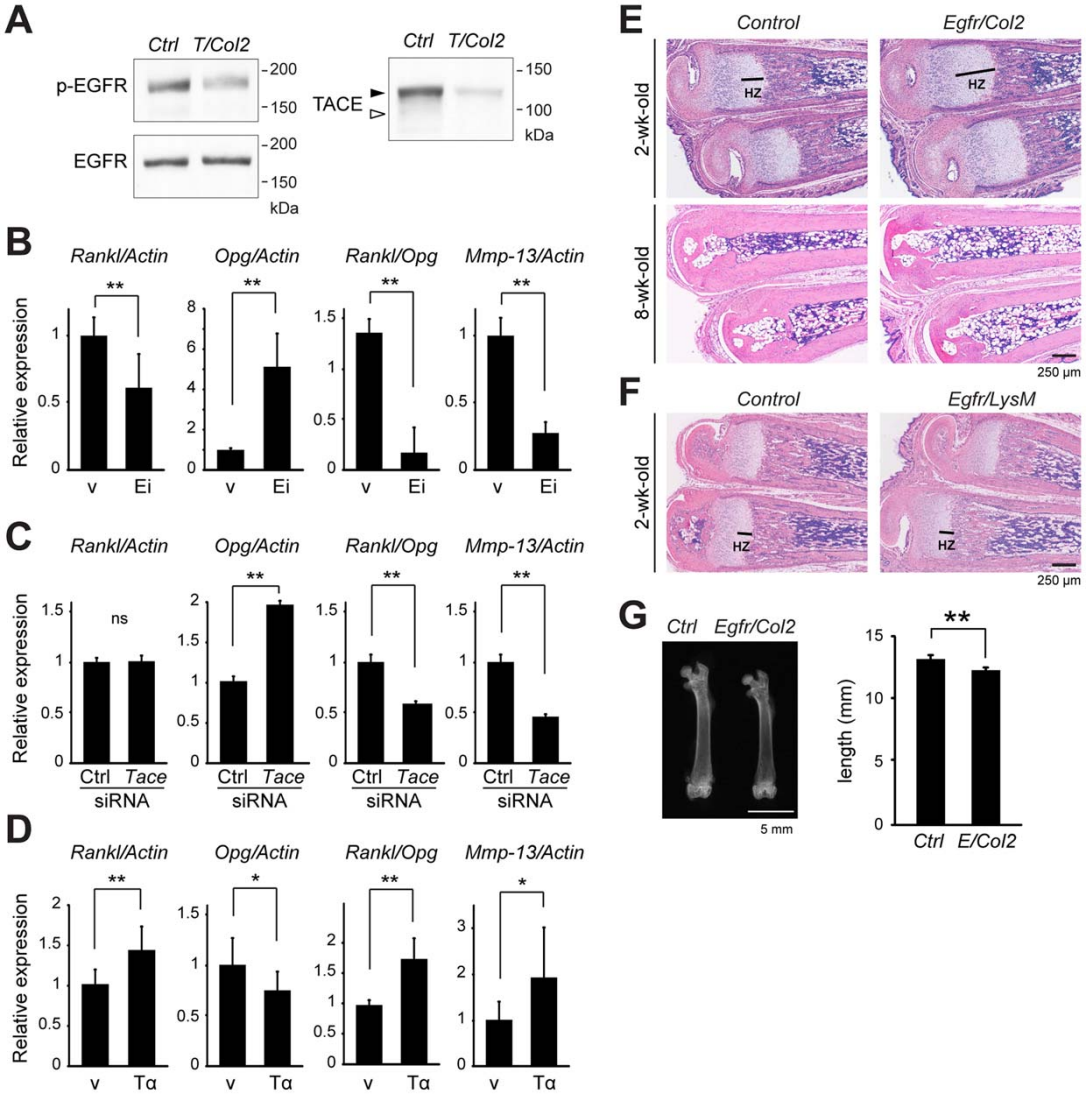
The conditional ablation of EGFR in chondrocytes leads to elongated growth plate and shorter long bones. (A) Western blot analysis of phospho-EGFR, EGFR, and TACE in the cartilage tissues collected from *Control* and *Tace/Col2* mice. (B–D) Primary chondrocytes collected from wild-type newborn mice were incubated with vehicle (v), EGFR inhibitor (Ei), or TGFα (Tα, 50 ng/ml) (B and D), or treated with control (Ctrl) or *Tace*-siRNA (C). The levels of the *Rankl*, *Opg*, *Mmp-13*, and β-Actin transcripts were analyzed by quantitative RT-PCR. The results are expressed as the mean ± SD of at three independent experiments. (E and F) Sections of the metatarsi from *Egfr^flox/flox^/Col2-cre* (*Egfr/Col2*), *Egfr^flox/flox^/LysM-cre* (*Egfr/LysM*), and *Control* mice. HZ, hypertrophic zone. (G) X-ray analysis of the femur of 8-week-old *Control* (*Ctrl*) and *Egfr^flox/flox^/Col2-cre* mice (*Egfr/Col2*). The results are expressed as the mean ± SD of at least three mice in each group. *, *p*<0.05. **, *p*<0.005.

Gene expression analysis of the cartilage tissues indicated that *Tace/Col2* mice are potentially defective in the recruitment of osteoclasts to the chondro-osseous junction and in the degradation of calcified cartilage due to a dysregulated expression of *Rankl*, *Opg*, and *Mmp-13*. Because TACE is a critical regulator of EGFR ligand activation *in vivo* [9], [13], [25], we asked whether EGFR signaling in chondrocytes is involved in the regulation of the expression of these genes. We first performed Western blot analysis using the cartilage tissues harvested from 1-week-old *Tace/Col2* and *Control* mice, and found that phosphorylation of EGFR was significantly reduced in the *Tace/Col2* cartilage compared with that in the wild-type control (Fig. 4A). This observation further underscores TACE as an essential regulator for EGFR signaling, and indicates that EGFR signaling is suppressed in the cartilage tissues lacking TACE. Given these observations, we next examined how the inhibition or activation of EGFR signaling would affect the regulation of *Rankl*, *Opg*, and *Mmp-13* transcripts in chondrocytes. As shown in Fig. 4B, we found that the inhibition of EGFR signaling using an EGFR inhibitor in primary chondrocytes results in a suppression of *Rankl* and *Mmp-13*, and an increase in *Opg* transcripts, in a similar manner as observed in the cartilage tissues lacking TACE (Fig. 3D). In accordance, gene silencing of *Tace* using siRNA enhanced *Opg* and suppressed *Mmp-13* transcripts; whereas no significant change was observed in the expression of *Rankl* transcripts (Fig. 4C). The reason for this discrepancy in not clear; nevertheless, the *Rankl*/*Opg* ratio was significantly reduced in the *Tace*-siRNA treated chondrocytes. Moreover, stimulation of EGFR with soluble TGFα showed a nearly opposite effect on the regulation of these genes to that with EGFR suppression (Fig. 4D). Taken together, these data indicate that the TACE-EGFR pathway in chondrocytes enhances osteoclast recruitment and matrix degradation thorough regulating RANKL, OPG, and MMP-13 expression.

### The disruption of EGFR in chondrocytes, but not in monocytes or macrophages, phenocopies the skeletal defects observed in *Tace/Col2* mice

To confirm the hypothesis that skeletal defects in *Tace/Col2* mice are derived from defective EGFR signaling in chondrocytes, we generated two different EGFR conditional knockout mice. We mated *Egfr^flox/flox^* mice [26] with *LysM-cre* transgenic mice [27], in which *cre* is specifically expressed in a macrophage-lineage cell population, to ablate EGFR in osteoclast-precursors (*Egfr^flox/flox^/LysM-cre*). To inactivate EGFR in chondrocytes, we also mated *Egfr^flox/flox^* mice with *Col2a1-cre* transgenic mice (*Egfr^flox/flox^/Col2-cre*). As, shown in Fig. 4E, we found that the chondrocyte-specific disruption of EGFR resulted in an elongated hypertrophic zone in young mice, which was reminiscent of the defects observed in the *Tace/Col2* mice. Note that the elongation of the growth plate was not as significant as observed in *Tace/Col2* mice, presumably due to an inefficient excision of *Egfr* alleles in *Egfr^flox/flox^/Col2-cre* mice as previously described [28], [29]. Furthermore, the elongation of the growth plate was transient during postnatal development and was not evident in the adult mutant mice. On the contrary, we found no skeletal defects in *Egfr^flox/flox^/LysM-cre* mice (Fig. 4F) or in *Tace^flox/flox^/LysM-cre* mice [17], [30] (data not shown), suggesting that the EGFR ligands released from cartilage cells in the growth plate act in an autocrine or paracrine manner and not as a chemoattractant for osteoclasts and their precursors. Furthermore, X-ray analysis revealed that the long bones of mutant mice that specifically lack EGFR in the chondrocytes are shorter than those of their wild-type littermates at 8 weeks of age (Fig. 4G). This was also consistent with the shortened long bones observed in the *Tace/Col2* mice (Fig. 1C and D). Taken together, these observations show that the conditional inactivation of EGFR in chondrocytes phenocopies the skeletal defects observed in *Tace/Col2* mice, indicating that the defective TACE-EGFR signaling axis in chondrocytes is responsible for the skeletal defects observed in *Tace/Col2* mice.

## Discussion

The present study shows that the chondrocyte-specific inactivation of TACE results in an elongated growth plate during postnatal long bone development. This defect is transient and becomes less obvious as the mice become older. By 8 weeks of age, the histological difference between *Control* and *Tace/Col2* animals is nearly undetectable; however, X-ray analysis shows that *Tace/Col2* mice exhibit shorter long bones than their wild-type littermates. In addition, there were no overt defects in the articular joints under unchallenged conditions even in older mice (data not shown).

Although TACE-deficient chondrocytes in the growth plate did not have apparent defects in cell proliferation or differentiation, there was a significant decrease in the number of TRAP-positive multinucleated osteoclasts adjacent to the chondro-osseous junction in the *Tace/Col2* mice compared to *Control* mice. Of note, we found that the *Tace/Col2* cartilage exhibited lower *Rankl* and higher *Opg* transcripts compared with those in the control littermates, indicating that chondrocytes lacking TACE are defective in the recruitment of osteoclasts to the calcified cartilage. Because TACE is essential for the activation of several EGFR ligands, and phosphorylation of EGFR was suppressed in the *Tace/Col2* cartilage, we examined how the inhibition of EGFR signaling would affect the expression of these genes. We found that treatment with a small-molecule EGFR inhibitor decreases the expression of *Rankl* and increases the expression of *Opg* in primary chondrocytes. Consistent with this observation, we also found that mutant mice lacking EGFR in the chondrocytes have skeletal defects similar to those in the *Tace/Col2* mice. There are seven different EGFR ligands in mammals, and at least five of them (TGFα, heparin-binding EGF-like growth factor, amphiregulin, epiregulin, and epigen) are highly dependent on TACE for their functional activation [13], [31]. Interestingly, it was recently shown that TGFα-null mice exhibited skeletal defects similar to those observed in the *Tace-Col2* mice (shorter long bones and an expansion of the hypertrophic zone) [32], indicating that lack of EGFR signaling driven by TGFα is involved in the defects observed in *Tace-col2* mice. In accordance, we also found that TGFα had a nearly opposite effect on the regulation of the transcription of *Rankl* and *Opg* in chondrocytes to those with EGFR inhibitor. Taken together, the present study suggests that EGFR signaling in chondrocytes positively regulates osteoclast recruitment, and that defective signaling in the TACE-TGFα-EGFR pathway in chondrocytes as the major cause for the phenotype of *Tace-Col2* mice. On the other hand, because TACE has diverse target substrates, and the defect in the growth plate is transient, it is likely that compensatory or redundant mechanisms exist in osteoclast recruitment and degradation of calcified cartilage matrix at the chondro-osseous junction.

While we were preparing the manuscript for the present study, another study describing the effects of gefitinib (Iressa) [33], a small-molecule inhibitor of EGFR, on postnatal skeletal development in rats and the skeletal phenotype of mutant mice in which EGFR expression was suppressed in chondrocytes was published [28]. Although that study did not include any data on the skeletal phenotype in adult animals (e.g., the length of long bones, etc.), the growth plate defects in their rat and mouse models were highly similar to those observed in *Tace/Col2* mice. Importantly, despite the differences in our animal models, our group and Zhang et al. [28] both concluded that the elongated hypertrophic zone in the growth plate was not a consequence of defects in chondrocyte proliferation or differentiation but was indirectly caused by a change in the equilibrium between OPG and RANKL, which results in failure to recruit osteoclasts to the calcified cartilage. This conclusion is also supported by a previous study that demonstrated that *Egfr^−/−^* embryos exhibit delayed primary endochondral ossification due to defective osteoclast recruitment to the calcified cartilage [34]. The observations in the present study and in the study by Zhang et al. [28] differ from those in Wang et al. [34] and Yi et al. [35], which suggest that EGFR ligands act directly on osteoclasts to affect their function and development. We found that mutant mice lacking EGFR in chondrocytes, but not those lacking EGFR in monocyte or macrophage cells, showed similar defects in the growth plate; this observation suggests that EGFR ligands are not direct mediators of osteoclast function.

Similar defects in the growth plate (delayed primary endochondral ossification, elongated hypertrophic zone, and shorter long bones) have been described in MMP-13-deficient [23], [24] and MMP-9-deficient mice [20]. In the developing bone, MMP-13 is highly expressed in hypertrophic chondrocytes and osteoblasts and is involved in the degradation of hypertrophic cartilage, whereas MMP-9 is predominantly expressed in osteoclasts. As shown in a previous study [28], we also found that EGFR signaling in chondrocytes positively regulates the expression of *Mmp-13*, and that *Mmp-13* transcripts expression is reduced in the *Tace/Col2* cartilage, indicating that elongation of the hypertrophic zone in *Tace/Col2* mice was, at least to some extent, derived from a repression of MMP-13 activity. In all of these mutant mice, the elongation of the hypertrophic zone was caused by a defective degradation of calcified cartilage and not by developmental defects in the chondrocytes *per se*. Theoretically, delayed degradation of calcified matrix could also lead to a delay in the subsequent recruitment of osteoblasts and bone formation, and it is tempting to speculate that this delay in the turnover of the growth plate results in shorter long bones. Nevertheless, the exact cause-and-effect mechanisms between the elongated hypertrophic zone and shortened long bones observed in these mutant mice should be studied further.

**Figure 5:**
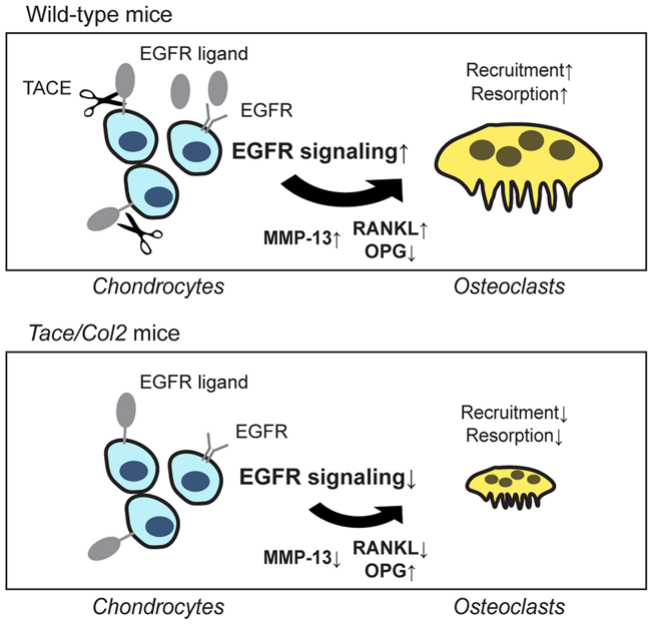
A proposed schematic model of the present study. The TACE-EGFR pathway in chondrocytes regulates the expression of RANKL, OPG, and MMP-13, so as to stimulate osteoclast recruitment and matrix degradation (upper panel). This mechanism is defective in the chondrocytes lacking TACE (lower panel).

In summary, we showed that the ablation of TACE in chondrocytes results in shorter long bones and elongation of the hypertrophic zone. Unexpectedly, there were no obvious defects in the differentiation or proliferation of TACE-deficient chondrocytes; however, the absorption of calcified cartilage was significantly delayed most likely due to the insufficient recruitment of osteoclasts to the chondro-osseous junction and reduced expression of MMP-13 (Fig. 5). Although the functions of TACE as a critical mediator of inflammation have been widely investigated [36], [37], the potential roles of TACE in skeletal development and skeletal disorders remain poorly understood. In this regard, this study may shed light on both ectodomain shedding and skeletal development.

## Materials and Methods

### Mice

The generation of *Tace^ flox/flox^* mice, *Egfr ^flox/flox^* mice, *Col2a1-cre* transgenic mice, and *LysM-cre* transgenic mice has been described previously [17], [19], [26], [27]. The mice were housed under specific pathogen-free conditions. All of the mice used in this study were of a mixed genetic background (129Sv, C57Bl/6), and all of the comparisons described here were made between littermates. All of the animal experiments in this study were approved by the Institutional Animal Care and Use Committee of the Keio University, School of Medicine (Permit Number: 09101-(5)). The mice were sacrificed by cervical dislocation under general anesthesia with ether.

### Histology

Tissue samples were fixed in 4% paraformaldehyde/PBS, paraffin-embedded, sectioned and then stained with hematoxylin and eosin or alcian blue solution. For BrdU labeling, BrdU (30 μg/g body weight) was intraperitoneally injected 3 hours prior to sacrificing the animal. Anti-MMP-9, anti-type-X collagen and anti-BrdU antibodies were obtained from R&D Systems, LSL, and Dako, respectively. The sections were imaged with a DS-Ri1 camera (Nikon, Tokyo, Japan) on a BX53 microscope (Olympus, Tokyo, Japan).

### Primary chondrocytes and quantitative RT-PCR

Primary chondrocytes were harvested from newborn mice as previously described [38], with some modifications. The chondrocytes were incubated with DMEM/F12 medium containing antibiotics and 5% fetal bovine serum for 4–5 days prior to the experiments. The cells were then plated in 12-well plates and incubated with or without EGFR inhibitor (10 μM, Cyclopropanecarboxylic acid-(3-(6-(3-trifluoromethyl-phenylamino)-pyrimidin-4-ylamino)-phenyl)-amide, Calbiochem) for 24 h. In a separate experiment, the chondrocytes were incubated with serum-free medium overnight and then incubated in the presence of human recombinant TGFα (Calbiochem) for 24 h. To examine the effect of TACE inhibition, *Tace*-siRNA (Dharmacon) introduced chondrocytes were incubated with serum-free medium for 24 h. At the end of the incubation, RNA was extracted using RNAiso (Takara Bio, Shiga, Japan) and reverse-transcribed using ReverTra Ace (Toyobo, Osaka, Japan). PCR amplification and quantification were performed using SYBR premix EX Taq II (Takara Bio) and LightCycler II (Roche). The relative mRNA expression levels were obtained by normalizing the samples to the expression level of β-Actin transcripts. The sequences of the oligonucleotides used in this study are available upon request.

### Western blotting

Primary chondrocytes, or cartilage tissues collected from the distal femur and proximal tibia from 7-days-old *Tace/Col2* mice or control littermates were lysed in 1% TritonX-100 in PBS with 1 mM 1,10 phenanthroline, protease inhibitor cocktail, and phosphatase inhibitor cocktail (Sigma-Aldrich). The lysed cartilage tissues were further incubated with Concanavalin A sepharose beads (Wako Chemicals, Osaka, Japan) to purify the glycosylated proteins. The samples were separated by SDS-polyacrylamide gels and transferred to nitrocellulose membranes. TACE was detected using anti-sera against the cytoplasmic domain of TACE [39]. The anti-β-Actin antibody was purchased from Sigma-Aldrich. The anti-phospho-EGFR and Anti-EGFR antibodies were purchased from Cell Signaling Technology (#3777 and #4267, respectively).

### Histomorphometric analysis

All of the histomorphometric analyses were performed in 8 week-old *Control* and *Tace/Sox9* littermates. The femurs were excised, fixed with 75% ethanol, embedded in glycol methacrylate resin, and sectioned into 3 μm slices. For double labeling, the mice were subcutaneously injected with calcein (8 mg/kg body weight) at 1 and 4 days before sacrifice. The sections were stained with toluidine blue and subjected to histomorphometric analyses under a light microscope with a micrometer using a semiautomated image analyzer (Osteoplan II, Carl Zeiss, Thornwood, NY). The parameters for the trabecular bone were measured in an area of 1.62–2.34 mm^2^ at 1.2 mm above the growth plate at the distal metaphysis. Four mice from each group were analyzed.

### Statistical Analysis

All of the experiments were conducted in triplicate. Standard deviations were calculated using Microsoft Excel. A student's t-test for two samples that assumed equal variances was used to calculate the P-values. P-values <0.05 were considered statistically significant.
